# The ecology of nasal colonization of *Streptococcus pneumoniae*, *Haemophilus influenzae *and *Staphylococcus aureus*: the role of competition and interactions with host's immune response

**DOI:** 10.1186/1471-2180-10-59

**Published:** 2010-02-23

**Authors:** Elisa Margolis, Andrew Yates, Bruce R Levin

**Affiliations:** 1 Department of Biology, Emory University, Atlanta, GA 30322, USA

## Abstract

**Background:**

The first step in invasive disease caused by the normally commensal bacteria *Streptococcus pneumoniae*, *Staphylococcus aureus *and *Haemophilus influenzae *is their colonization of the nasal passages. For any population to colonize a new habitat it is necessary for it to be able to compete with the existing organisms and evade predation. In the case of colonization of these species the competition is between strains of the same and different species of bacteria and the predation is mediated by the host's immune response. Here, we use a neonatal rat model to explore these elements of the ecology of nasal colonization by these occasionally invasive bacteria.

**Results:**

When neonatal rats are colonized by any one of these species the density of bacteria in the nasal passage rapidly reaches a steady-state density that is species-specific but independent of inoculum size. When novel populations of *H. influenzae *and *S. pneumoniae *are introduced into the nasal passages of neonatal rats with established populations of the same species, residents and invaders coexisted. However, this was not the case for *S. aureus *- the established population inhibited invasion of new *S. aureus *populations. In mixed-species introductions, *S. aureus *or *S. pneumoniae *facilitated the invasion of another *H. influenzae *population; for other pairs the interaction was antagonistic and immune-mediated. For example, under some conditions *H. influenzae *promoted an immune response which limited the invasion of *S. pneumoniae*.

**Conclusions:**

Nasal colonization is a dynamic process with turnover of new strains and new species. These results suggest that multiple strains of either *H. influenzae *or *S. pneumoniae *can coexist; in contrast, *S. aureus *strains require a host to have no other *S. aureus *present to colonize. Levels of colonization (and hence the possible risk of invasive disease) by *H. influenzae *are increased in hosts pre-colonized with either *S. aureus *or *S. pneumoniae*.

## Background

The first step in a bacterial disease is the successful establishment of a bacterial population in a host: colonization. The conditions that determine whether a bacterial population can colonize a particular site and the density achieved are fundamental to determining the likelihood of invasive disease, transmission to other hosts and the presence of mutants resistant to antibiotics. How these conditions are affected by prior colonization by bacteria of the same or different species has wide spread consequences for determining the sequelae of the wide-scale use of vaccines directed at specific strains or species (as the vaccine strain/species can potentially be replaced by other potentially invasive strains and species [1]) as well as for evaluating probiotics [2] and understanding epidemiological changes in invasive bacterial diseases [3,4].

Whether bacteria can colonize or not is determined by many ecological factors including the availability of resources (i.e. nutrients, space, attachment space), host immune responses and the presence of toxins or harmful substances. As the presence of established bacteria populations can influence all of these factors, it seems reasonable to assume that co-inhabitants often determine whether colonization can occur. In fact co-inhabitants that are ecologically similar, should limit the colonization as the one that is better at exploiting the habitat should exclude the others through resource limitation [5]. However, as a consequence of even subtle differences in resource (ie nutrients, space or metabolic byproducts) utilization or availability, multiple strains and species of bacteria can co-exist [6-12]. The ability to colonize can also be influenced by interference, which includes residents populations producing harmful substances (like bacterocins [13,14]) or inducing an immune response [15,16]. In the case of three bacterial species which colonize the human nasopharynx (*Streptococcus pneumoniae*, *Staphylococcus aureus *and *Haemophilus influenzae*), epidemiological studies show that co-colonization is rarer than expected [17-21]. These co-inhabitation patterns suggest that there may be interference or competition occurring.

In this report we apply an ecological framework to elucidate the factors contributing to the nasal colonization of neonatal rats of three bacterial species that typically colonize humans: *S. pneumoniae*, *H. influenzae *and *S. aureus*. First we consider the population dynamics of each strain separately. We provide evidence that all three species colonize the nasal passages of neonatal rats and reach an apparent steady-state density and that this level is independent of inoculum density. To explore the effects of co-inhabitants on colonization, 48 hours after colonizing neonatal rats with one species we pulsed with a second inoculum of a marked strain of the same species. The results of these pulse experiments suggest that resident *S. aureus *prevents co-colonization of the same strain; while for both *H. influenzae *and *S. pneumoniae *the total density is increased to allow for the co-existence of pulsed and established populations. We repeated these experiments with the resident and invading populations being of different species and found that *H. influenzae *colonizes at a higher density when either *S. aureus *or *S. pneumoniae *are present and that immune-mediated competition between *S. pneumoniae *and *H. influenzae *is both site and strain specific.

## Results and Discussion

### Population Dynamics

**Figure 1:**
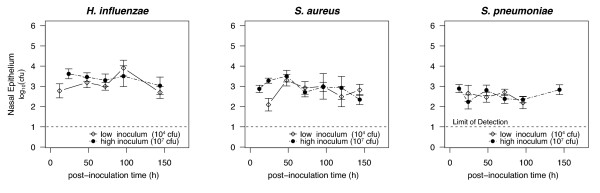
**Population dynamics of nasal colonization**. Population dynamics of nasal colonization. Five-day-old neonatal rats were inoculated with 10^7 ^(black circles) or 10^4 ^cfu (diamonds) of either *S. pneumoniae*, *H. influenzae *or *S. aureus*. The geometric mean bacteria density in the nasal epithelium of 4-16 rats at each time-point is plotted. Dashed line represents limit of detection. Error bars represent SE.

All three species readily colonize the nasal passages of neonatal rats. Within 48 hours after one of the three species is inoculated, *H. influenzae*, *S. aureus *and *S. pneumoniae *reach and maintain for at least three days a constant population (between 100-10,000 cfu depending on the species) in the nasal epithelium (Figure 1). The population dynamics of nasal colonization did not differ in the nasal wash sample with the nasal epithelium. The nasal epithelium, which represent the persisting colonizing bacteria [22], data are shown.

**Figure 2:**
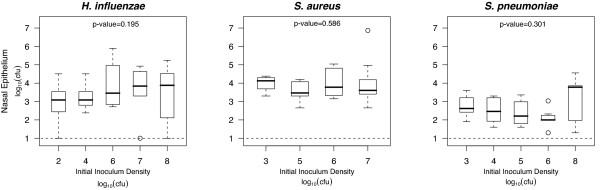
**Bacterial load is independent of inoculum density**. Groups of 7-16 five-day-old neonatal rats were inoculated with 10^2^-10^8 ^cfu of either *S. pneumoniae*, *H. influenzae *or *S. aureus*. The 25th to 75th percentiles of nasal wash and epithelium samples taken 48 hours after bacterial challenge are represented by the box plots, with the bold horizontal bar indicating the median value, circles outlying values and dotted error bars SE. *P *values were determined by Kruskal-Wallis rank sum which tested the null hypothesis that the bacterial load are distributed the same in all of the inoculum groups. Dashed line represents limit of detection.

The bacterial load for each of the species was not significantly different from 48 to 96 hours (p-values for each species determined by Kruskal-Wallis rank sum were < 0.05). While the dynamics for both a low and high inoculum density appear to be similar, we ascertained whether bacterial load is inoculum-independent at 48 hours after inoculation. For all three species the bacterial load is invariant over a wide range of inocula (10^2^-10^8 ^cfu) (Figure 2), suggesting that nasal colonization rapidly reaches a steady-state that is not limited by how many bacteria are inoculated.

### Invasion of Same Species in a Colonized Host

To test whether nasal colonization can occur in the presence of the same species, new populations of bacteria were pulsed (10^4 ^cfu inoculated) into rats that were already colonized by bacteria of that species. Antibiotic markers that conferred no in vitro or in vivo fitness costs were used to distinguish the resident and pulsed populations and each experiment was repeated reversing the strains as pulsed or resident to control for any fitness differences. As the population dynamics suggest that the bacterial load for each of these species is tightly controlled, we expected that the total density (resident+pulsed) would return to the bacterial load observed in rats without pulses. Because resident and pulsed strains of the same species utilize the same resource (and attract the same immune responses), co-existence of both strains is expected unless a limiting factor is available only on a first come first serve basis.

**Figure 3:**
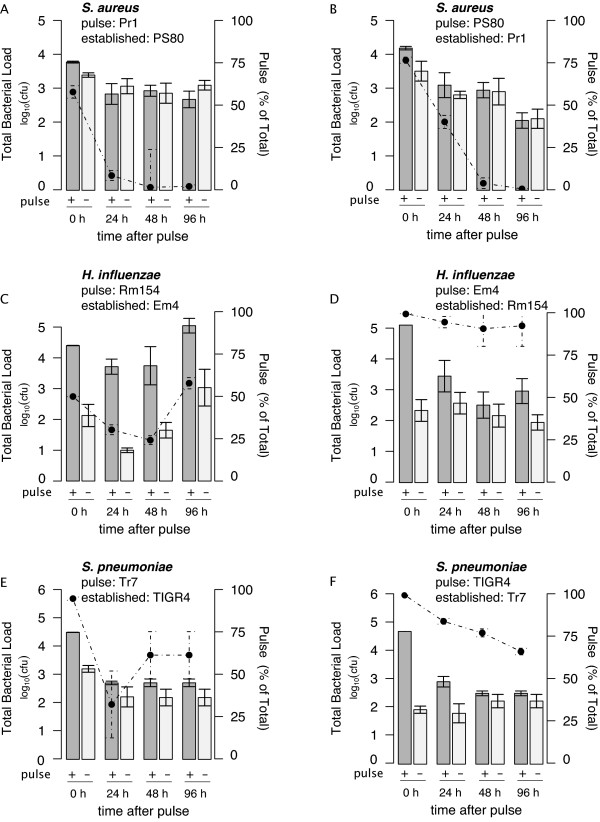
**Pulse on established populations of same species**. Established populations were inoculated into 3-day-old neonatal rats 48 hours prior to pulsing 10^4 ^cfu of a marked strain of the same species or PBS. The total bacterial density in nasal epithelium of 6-8 rats with the established and pulsed population (dark grey) and just the established population (light grey) were tracked over 96 hours after the pulse and expressed as the geometric mean with error bars indicating SE. In addition, the percent of the bacterial density that is pulsed is marked with points with dotted error bars indicating SE. Antibiotic marked strains were switched to be either pulsed or established for *H. influenzae *(in A and B), *S. aureus *(in C and D) and *S. pneumoniae *(in E and F).

In the case of *S. aureus*, regardless of whether the marked strain is resident or pulsed, we find that the pulsed strain declines in density (faster relative to the established) over the course of 96 hours (as shown in representative experiments in Figure 3A-B). As the pulsed strain declines (decrease in percent shown in dotted line) the total bacterial load of *S. aureus *in the rats with the pulsed and established strain (+ pulse) doesn't differ from the total density of *S. aureus *in the rats with only the established strain (- pulse). For *S. aureus *the bacterial density does not exceed that observed in rats without a pulse and the resident strain has a competitive advantage.

For both *S. pneumoniae *and *H. influenzae *there is an increase in the total density in the rats with the pulse (+ pulse) compared to rats with only the established strain (as shown in representative experiments in Figure 3C-F). We saw the bacterial load increase to varying degrees, more so for *H. influenzae *than for *S. pneumoniae*, in each of four replicate experiments (data available upon request). In both of these species, we observe that the pulsed and resident strains co-exist with the pulse strain becoming 25-90% of the population.

For all the species, similar pulse results were obtained in reciprocal experiments (switching pulse and resident strains) confirming that the results were not due to fitness differences in the antibiotic marked strains.

### Invasion of Different Species in a Colonized Host

Competition between different strains or species can be defined simply as a reduction in the density of one or both strains when both are present. Competition within the same species and particularly in the case of the same strain (as in the above pulse experiment) is usually mediated through a limiting shared resource. Competition between species, in addition to partitioning of a shared resource, can be mediated through inhibitory agents/toxins (allelopathy) or predators (in this case components of the immune system [23]). Previous studies suggest that production of hydrogen peroxide by *S. pneumoniae *may affect the densities of other species [24,25] and that immune-mediated competition reduces *S. pneumoniae *density in the presence of *H. influenzae *[26]. To evaluate the contributions of these different competitive mechanisms we performed invasion experiments (with one strain of each species: Eagan, TIGR4 and PS80) in which one species was resident and a second was introduced (an invader).

#### Evidence for synergistic interactions between *H. influenzae *and *S. pneumoniae *or *S. aureus*

**Figure 4:**
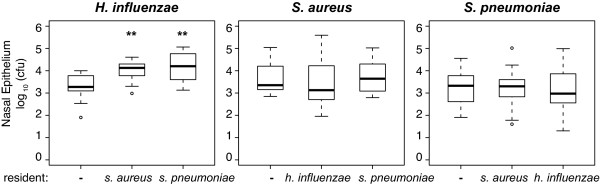
**Invasion of a host colonized with another species**. Established populations were inoculated into groups of 10-22 three-day-old neonatal rats 48 hours prior to pulsing 10^5 ^cfu of a different species or PBS. The 25th to 75th percentiles of nasal wash and epithelium samples taken 48 hours after bacterial challenge are represented by the box plots, with the bold horizontal bar indicating the median value, circles outlying values and dotted error bars. T-test *P *values < 0.005 are represented by **. Resident bacterial density was not significantly different from un-invaded rats in any combination of species.

Rather than an antagonistic interaction, we found that *H. influenzae *reached a higher density when invading resident populations of either *S. aureus *or *S. pneumoniae *than in the absence of these residents (Figure 4). A similar increase in the bacterial density of *H. influenzae *was observed *in vitro*; when mixtures of these strains were grown in broth for 6 hours, *H. influenzae *density was 20%(± 14) greater with *S. pneumoniae *and 19%(± 3) greater with *S. aureus *present than when grown alone (data not shown).

#### Strain-specific, innate immune-mediated interactions between *H. influenzae *and *S. pneumoniae*

We had expected to detect immune-mediated competition between *H. influenzae *and *S. pneumoniae*, as observed in a mouse model of colonization by Lysenko and colleagues [26]. However, we saw no evidence of competition between *H. influenzae *and *S. pneumoniae *with the strains we initially used: TIGR4 and Eagan (Figure 4).

**Figure 5:**
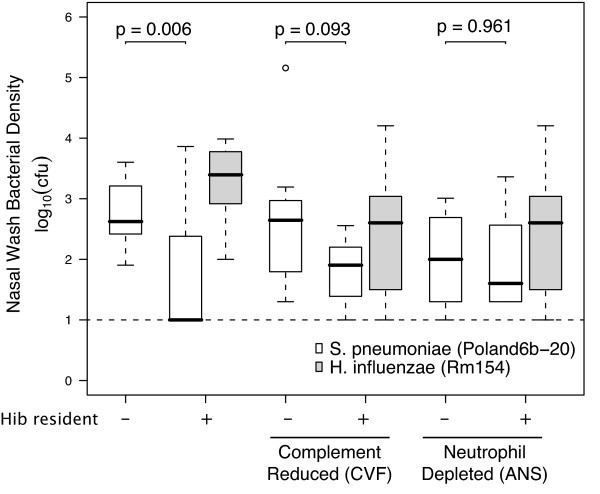
**Neutrophil- and Complement- Mediated Competition**. Three-day-old neonatal rats were treated with either anti-neutrophil serum (-neutrophil) or cobra venom factor (-complement) or PBS and inoculated with either 10^6^cfu of *H. influenzae *or PBS (alone). Forty-eight hours later, 10^4 ^cfu of Poland(6b)-20 *S. pneumoniae *was inoculated. The 25th to 75th percentiles of nasal wash samples taken 48 hours after *S. pneumoniae *inoculation are represented by the box plots, with the horizontal bar indicating the median value and circles outlying values. *P*-value from Mann Whitney U test comparing the bacterial density of previously uninfected rats and those with established populations of *H. influenzae*. Dashed line represents limit of detection.

To investigate further, we tested one additional strain of *S. pneumoniae*, Poland(6b)-20. We found that this particular strain of *S. pneumoniae *had a reduced density in the nasal wash, but not the nasal epithelium, when invading in a neonatal rat with an established *H. influenzae *population (Figure 5). This reduction in Poland-20's population did not occur in neonatal rats which had been depleted of complement or neutrophils.

**Figure 6:**
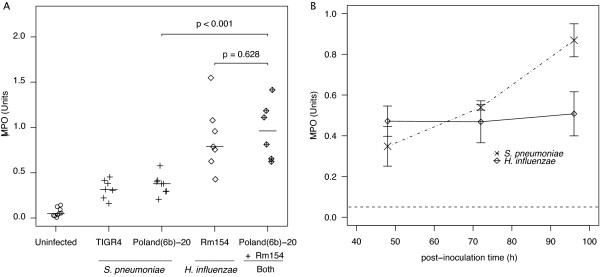
**Neutrophil infiltration: comparison of strains and species at 48 hours and dynamics over 96 hours**. A) Neutrophils in the nasal epithelium from rats inoculated 48 hours earlier with 10^4 ^cfu of bacteria from a single species (Rm154, TIGR4 and Poland(6b)-20) or from rats inoculated 96 hours earlier with 10^6 ^cfu of *H. influenzae *and 48 hours earlier with 10^4 ^cfu of Poland(6b)-20 were quantified using the MPO assay. Lines indicate median MPO values. *P*-value is calculated by the Wilcoxon rank sum test. B) Dynamics of neutrophil infiltration in response to nasal colonization by *S. pneumoniae *(TIGR4) or *H. influenzae*. Following inoculation groups of 5-8 rats were sacrificed and neutrophil infiltration was measured by MPO assay. Median MPO Units are plotted. Error bars represent SE. Dashed line represents median MPO of uninoculated rats.

To explain why we could only observe this in one of the two strains tested and only then in the nasal wash, we hypothesized that either induction of or susceptibility to the immune response must differ in these strains and locations. We quantified the neutrophil infiltration in the nasal epithelium by measuring the Myeloperoxidase (MPO) activity at 48 hours after inoculation with each strain/species alone or when Poland(6b)-20 was inoculated on an established *H. influenzae *population (Figure 6A).

No difference in neutrophil infiltration is observed between rats colonized by the two different *S. pneumoniae *strains (TIGR4 and Poland(6b)-20). The neutrophil infiltration observed 48 hours after Poland(6b)-20 invaded on an established *H. influenzae *population (when immune-mediated competition was observed in the nasal wash) was significantly higher than rats with just Poland(6b)-20 colonizing alone. However, neutrophil infiltration was not significantly higher than in rats with only *H. influenzae*. While these results suggest that *H. influenzae *is primarily responsible for the neutrophil infiltration that reduces the nasal lumen populations of some strains of *S. pneumoniae*, *S. pneumoniae *may still have a role in eliciting the immune response (perhaps with slower dynamics than *H. influenzae*). We observed that the neutrophil infiltration in response to *S. pneumoniae *colonizing alone increases from 48-96 hours after inoculation, compared to the constant neutrophil presence with *H. influenzae *(Figure 6B).

## Discussion

### Population Dynamics

All three species that we studied (*S. aureus*, *S. pneumoniae *and *H. influenzae*) can colonize the nasal passages of neonatal rats and each reaches a bacterial load that is independent of the initial inoculum size; they increase in density when initially below this level and decline when initially above it. This indicates that the steady-state density is tightly controlled - perhaps by a limiting resource or the host's immune response. The total density of each of these colonizing species is relatively low and there is wide-spread variation in the densities of individuals, similar to what has been observed in colonized humans [27].

### Invasion of Same Species in a Colonized Host

In accord with classical ecological theory of competition [5] we would anticipate that established populations of one strain of a species would either prevent colonization of another strain of that species or be eliminated. The outcome would depend on which of the strains was more fit. In the case of genetically marked strains of the same species with equal fitness we would expect to observe co-existence. In a series of pulse experiments with genetically marked colonizing (pulsed) and resident (established) strains we found the situation to be more complicated than this simple interpretation.

For both *H. influenzae *and *S. pneumoniae*, the resident and the pulsed strains co-existed. Surprisingly in all replicates of these experiments, the invasion of a second population of the same species was followed by an increase in the total bacterial density. Given that the steady-state bacterial densities were independent of the initial inoculum density following a single inoculation, we had expected that the bacterial density after a second inoculation would decline to the original bacterial load. These results might be attributable to a second inoculation leading to an expansion in the colonization area, increased immune suppression or the release of new resources - perhaps associated with an inflammatory response.

On first consideration it would seem that the results of the *S. aureus *pulse experiments are consistent with classical ecological theory; the established population of this species inhibited the colonization of a new strain. Moreover, as expected following the pulse by a second strain the total density of *S. aureus *returned to a level similar to that observed in the single inoculation experiments. However the resident strain had the advantage no matter which marker it carried (the competitive exclusion observed was not due to difference in fitness between the marked strains). We interpret these results as suggesting that *S. aureus *is limited by a localized resource available on a 'first-come, first-serve' basis - perhaps attachment sites [28,29]. This ecological hypothesis would account for the observations that competing strains of *S. aureus *were excluded from burn wounds [30] and from nasal colonization in persistent human carriers [31]. While these results do not exclude the possibility that variation within *S. aureus *strains may allow for coexistence as occasionally has been observed in humans [32], they do suggest that prior nasal colonization with *S. aureus *can exclude similar *S. aureus *strains from colonizing.

### Invasion of Different Species in a Colonized Host

Ecologically, strains of different species would be anticipated to be more divergent than those of the same species and therefore they would be expected to occupy different niches. The results of our experiments are consistent with this interpretation as any pairwise combination of the three species can co-exist. While we had expected some sharing of resources by these different species, we found no evidence that the presence of one species reduced the density colonizing the nasal epithelium of another species.

The only evidence for inter-specific interactions between these species observed at the nasal epithelium was in fact synergistic rather than antagonistic; *H. influenzae *reaches a higher density when invading resident populations of either *S. aureus *or *S. pneumoniae *than it achieves in rats not colonized by these bacteria. Since this result is also observed in vitro it seems likely due to some host-independent mechanism such as *S. aureus *and *S. pneumoniae *providing nutrients that would otherwise limit *H. influenzae*. Indeed, in the past *H. influenzae *has been identified and cultured due to the fact that it grew as satellites off of *S. aureus *colonies [33]. To our knowledge this is the first evidence for *S. aureus *and *S. pneumoniae *increasing the density of *H. influenzae *during nasal colonization.

We had expected to see inter-specific antagonism not only due to resource sharing but also because of interference by toxins and harmful substances. In fact, it has been proposed that the production of hydrogen peroxide by *S. pneumoniae *may affect the densities of *S. aureus *and *H. influenzae *as both are susceptible to hydrogen peroxide killing [24,25,34,35]. However in this and a previous work that specifically addressed this issue [36] we found no evidence that hydrogen peroxide produced by *S. pneumoniae *limits the colonizing populations of either of the two species. This may be because the density of *S. pneumoniae *is too low for sufficient hydrogen peroxide production or the nasal epithelium inactivates the hydrogen peroxide produced. Taken at large, we found no ecological interaction between *S. aureus *and *S. pneumoniae *colonization that would account for the epidemiological observation that *S. aureus*-*S. pneumoniae *co-colonization is rarer than expected [4,18,20,37,38]. We postulate that this epidemiological observation may be due to the bacteria preferring different hosts rather than competitive interactions within hosts [39], or that competitive exclusion may only occur in immunologically mature individuals. Others have suggested that there may still be an ecological interaction based on the pneumococcal pilus [35] or by induction of phage release [40].

### Neutrophil-mediated Competition

Previous experiments by Lysenko and colleagues in a mouse model have shown that when *H. influenzae *and *S. pneumoniae *co-colonize, *S. pneumoniae's *density in the nasal wash is lower than when inoculated alone due to immune-mediated competition [26]. At one level, the results of our rat model experiments with *H. influenzae *and *S. pneumoniae *are consistent with their results [26]. However, our results also suggest that this immune-mediated competitive interaction may only affect the colonizing *S. pneumoniae *population in the nasal wash (not the population adhering to nasal epithelium) and is strain-specific. We observed immune-mediated competition with the clinical strain of *S. pneumoniae *Poland(6b)-20 but not with TIGR4. We hypothesize that pneumococcal strains vary either in their ability to elicit an immune response or in their suscebtibility to the immune response induced by *H. influenzae*. Although we can't exclude the possibility that the two strains we tested elicited different immune responses, our results suggest that there is no difference in the extent of neutrophil infiltration of the epithelium in response to colonization by either of these strains or any synergism [41] between the two species. Together our results suggest that the immune response primarily elicited by *H. influenzae *is responsible for reducing the density of *S. pneumoniae *in the nasal wash and that *S. pneumoniae *strains may vary in their susceptibility to this innate immune response. While we found limited evidence for immune-mediated competition, since the nasal epithelium bacterial populations of *S. pneumoniae *are un-altered by this innate immune response this competition may not effect the long-term carriage of *S. pneumoniae *in the nasal passage.

### Limitations

Perhaps the most significant limitation and caveat associated with this study is that the neonatal rat immune system is changing during the course of these experiments, thereby restricting our ability to draw inferences about the role of the immune response and long-term colonization dynamics. While arguably a decent model for young infants, the neonatal rats are unlikely to be an accurate model of the nasal passages of older children or adults. Another limitation of this study is that the results obtained may be strain-specific and only one or two strains for each species was tested. The limited number of strains does not likely reflect the within species diversity in colonization strategies and this diversity should be investigated in further studies. Finally, our ability to draw inferences about the factors influencing the ecology of colonization in these neonatal rats was limited by the substantial amount of variation in densities observed in individual rats.

## Conclusion

**Figure 7:**
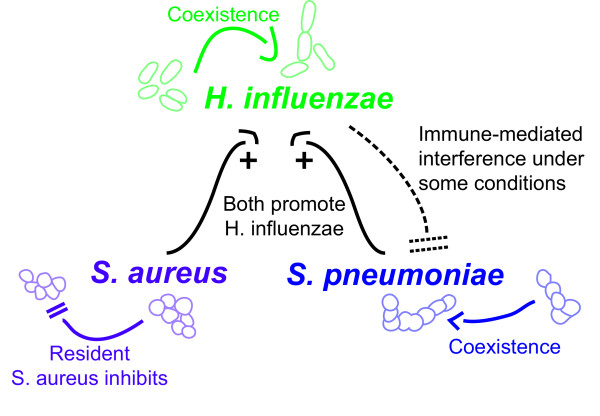
**Dynamic Process of Nasal Colonization**. Graphical interpretation of Pulse and Invasion Experiments.

Caveats and limitations aside, we believe that the application of an ecological framework to the colonization of neonatal rat model with *S. aureus*, *S. pneumoniae *and *H. influenzae *contributes to our understanding of the epidemiology of carriage, disease processes and the impact of vaccination on these bacteria species. These results begin to address the mechanisms responsible for the dynamic process of nasal colonization with turnover and replacement of species, serotypes and strains in the complex community (Figure 7). For example the pulse experiments results suggest that for *S. pneumoniae *and *H. influenzae *the presence (and turnover) of multiple strains and serotypes would be expected in carriers as has been observed in humans [42]. Further, our results suggest that that *H. influenzae *colonization will be more successful (and hence possibly more likely to cause disease) when preceded by either *S. aureus *or *S. pneumoniae*. Ultimately the ecology of nasal colonization informs whether vaccination (or antibiotic treatment) directed at one particular species will lead to the unintended consequences of increased colonization by competing (and possibly more pathogenic) species, serotypes or strains.

## Methods

### Bacterial strains, media and inoculum preparation

A laboratory bacterial strain of each species was selected based on capsular type and invasive potential. *S. pneumoniae *TIGR4 (serotype 4) [43] and Poland(6b)-20(serotype 6b) [44] were provided by Lesley McGee. Tr7 was selected as a spontaneous rifampicin resistant mutant of TIGR4. *S. aureus *PS80 (serotype 8 ATTC 27700) was obtained from American Type Culture and Pr1 was selected as a spontaneous mutant of PS80 exhibiting resistance to rifampin. *H. influenzae *type b Eagan and its streptomycin resistant mutant Rm154 were provided by Richard Moxon. Em4 was selected as a spontaneous mutant of Eagan exhibiting resistance to nalidixic acid.

*S. pneumoniae *strains were grown in Todd-Hewitt broth (Becton Dickinson) supplemented with 0.5% w/v of yeast extract (THY) and plates were supplemented with 4% v/v of sheep blood (BBL). Broth cultures and agar plates of *S. pneumoniae *were incubated at 37°C with 5% CO_2 _*H. influenzae *strains were grown in brain heart infusion broth (Becton Dickinson) supplemented with 10 *μ*g of hemin (sigma) and 2 *μ*g of *β*NAD (sigma) per ml (sBHI). *S. aureus *strains were cultivated in Luria-Bertani (LB; Becton Dickinson,) broth cultures.

Equal fitness of antibiotic marked strains was confirmed by mixing equal densities of cultures in exponential phase and sampling the initial densities and the densities 6 hours later in broth or 48 hours later in nasal passages of neonatal rats. For all combinations (i.e. TIGR4/Tr7, PS80/Pr1, Rm154/Em4), there was no significant fitness difference in vitro or in vivo (data not shown). The spontaneous antibiotic resistant mutant strains were repeatedly grown alone in broth and consistently showed 100% plating efficiencies when plated on media with antibiotics versus media alone.

To determine if synergistic interaction between *H. influenzae *occurred in vitro when co-cultured with either *S. pneumoniae *or *S. aureus*, *H. influenzae *was grown in sBHI with or without another species and the intial densities and the densities 6 hours later were compared.

Inoculum for all the infant rat experiments were prepared by initially growing strains to late logarithmic phase (*OD*_620_:0.35-0.8). These were stored at -80°C and then thawed before suspending in 2 ml of either LB, THY or sBHI. Mid-exponential phase cultures were centrifuged (5,000 g × 3 min) and resuspended in phosphate-buffered saline with 0.1% gelatin (PBSG). Note the addition of gelatin did not lead to an increase in the inoculation density for any of these bacteria. Bacterial densities were estimated by plating dilutions of *S. aureus *on LB Agar plates or LB plates supplemented with rifampicin (40 mg/L); *S. pneumoniae *on THY blood plates supplemented with either streptomycin (40 mg/L) or rifampicin (50 mg/L) or *H. influenzae *on sBHI plates supplemented with bacitracin (0.3 g/L) and either streptomycin (4 mg/L) or nalidixic acid (5 mg/L).

### Infant Rat Model

Although neonatal rats do not naturally carry *S. aureus*, *S. pneumoniae *and *H. influenzae*, they can be reproducibly colonized with these species. All animal experiments were performed under the guidelines approved by the Emory Institutional Animal Care and Use Committee. Three-day-old pups, born of timed-pregnant Sprague-Dawley rats (Charles River Laboratories), were randomly reassigned to dams. At 3 or 5 days of age, rats were intranasally inoculated by touching a drop of 10^2 ^- 10^8 ^bacteria of either *S. aureus*, *S. pneumoniae *or *H. influenzae *(that had been spun down and re-suspended in 5 *μ*l PBS supplemented with 0.1% gelatin (PBS-G)) to the right and then another 5 *μ*l to the left external nares [45]. The nasal flora of un-inoculated neonatal rats, determined by colony morphology on blood plates, appeared to consist primarily of non-hemolytic streptococci and coagulase-negative staphylococci. No *S. aureus*, *S. pneumoniae *and *H. influenzae *colonies were isolated from un-inoculated neonatal rats and all of these strains colonized in spite of the presence of this nasal flora.

Two days after the innoculation, nasal wash was collected from 200 *μ*l of PBS-G instilled into a 5 cm intramedic polyetylene tubing (PE50, intramedic, Clay Adams) placed into the trachea, and nasal epithelium was scraped from the nasal passages after a second wash of 200 *μ*l of PBSG and removal of the frontal bones. 3 sequential nasal washes of 200 *μ*l of PBS-G contained no significant decrease in the bacteria density compared to the first wash. The nasal epithelium was homogenized in 1 ml of PBS-G.

In all experiments, 100 *μ*l of the nasal wash and nasal epithelium samples were plated directly and serially diluted onto selective plates. The limit for detection was 10 cfu/ml. Nasal wash densities were converted to cfu in rat by multiplying cfu/ml by 5 (200 uL total vol.) and nasal epithelium by multiplying by 1 (1 ml total vol.). With the exception of the *H. influenzae *-*S. pneumoniae *interaction, data from the nasal wash and nasal epithelium data are in agreement and only the nasal epithelium data are presented; as nasal epithelium likely represents the persistent colonizing population [22].

### Experimental Design

For the population dynamics of nasal colonization, groups of 4-16 5-day-old rats were intranasally inoculated with either 10^4 ^or 10^7 ^cfu bacteria of *S. aureus*, *S. pneumoniae *or *H. influenzae *and sampled 12-144 hours after inoculation. Inoculum independence was confirmed by inoculating groups of 7-16 5-day-old rats with 10^2^- 10^8 ^cfu bacteria of *S. aureus*, *S. pneumoniae *or *H. influenzae *and sampling at 48 hours.

For intra-species invasion, one marked variant of a particular strain was intranasally inoculated into two groups of 24-36 3-day-old rats. Fourty-eight hours later one group was intranasally inoculated with the same species with the alternative antibiotic marker while the other inoculated with PBS. At 0, 24, 48 and 96 hours after pulsing with the same species 6-8 rats were sacrificed and sampled. For each pairing between antibiotic marked strains of the same species (i.e. TIGR4/Tr7, PS80/Pr1, Rm154/Em4), this experiment was repeated with the reverse strain being established and pulsed.

For the inter-species invasion, experiments testing, groups of 8-12 3-day-old rats were inoculated in both nostrils with either one species (*S. aureus*, *S. pneumoniae *or *H. influenzae*) or with PBS. All of these rats were then inoculated 48 hours later with 10^6^- 10^7 ^of another species (*S. aureus*, *S. pneumoniae *or *H. influenzae*), and then sacrificed 48 hours after the inoculation of second species.

### Immune Depletion

For systemic complement reduction, cobra venom factor (CVF; Advanced Research Technologies, San Diego, CA) was administered to 4-day-old neonatal rat by intraperitoneal injection of 500 *μ*g/kg of weight (dissolved in 0.1 M PBS) [46]. Systemic complement reduction was confirmed by the EZ Complement CH50 Test kit (Diamedix, Miami, FL) [47]. Serum from age matched un-inoculated control rats had CH50 of 40.94 ± 6.6, while CVF treated rats had a CH50 of 21.6 ± 3.9 until 5 days after CVF treatment.

For systemic neutrophil depletion, anti-neutrophil serum (ANS, absorbed rabbit anti-rat PMN; Accurate Chemical, Westbury, NY) was administered to 4-day-old neonatal rat by subcutaneous injection of 6 *μ*L/g of weight (diluted 1:1 in PBS) [48]. Systemic neutrophil depletion was confirmed by FACS analysis of blood and local depletion confirmed in the nasal passages using a myeloperoxidase (MPO) assay of nasal epithelium [49]. In ANS treated un-inoculated rats nasal epithelium MPO was 0.002 ± 0.01 U, compared to control rats 0.072 ± 0.02 U.

### Statistical Analysis

The bacterial densities (and the *log*_10 _transformed densities) during colonization were not normally distributed. To determine whether inoculum size altered the median bacterial density or whether the density varied from 48 to 96 hours post-inoculation, a Kruskal-Wallis rank sum test was used to compare the ranks for each inoculum size or time point. A Wilcoxon rank-sum test was used to evaluate the statistical significance in inter-species competitions or the myeloperoxidase results for different strains.

## Authors' contributions

EM conceived of, undertook and analyzed all of the experiments. AY assisted in the conception and analysis of the pulse experiments. BRL was a supportive kibitzer and advised the conception and interpretation of all the experiments. All three authors contributed to the writing of this manuscript.
